# High-Throughput Proteomic and Glycoproteomic Analyses in Benign Prostatic Hyperplasia

**DOI:** 10.1021/jasms.6c00143

**Published:** 2026-09-02

**Authors:** Feixuan Wu, Alexis E. Adrian, Allen Zhao, Han Zhang, Peng-Kai Liu, Yajing Lu, Douglas W. Strand, William A. Ricke, Lingjun Li

**Affiliations:** School of Pharmacy, University of Wisconsin-Madison, Madison, Wisconsin 53705, United States; Department of Urology, George M. O’Brien Center of Research Excellence, University of Wisconsin-Madison, Madison, Wisconsin 53705, United States; Department of Biochemistry, University of Wisconsin-Madison, Madison, Wisconsin 53706, United States; School of Pharmacy, University of Wisconsin-Madison, Madison, Wisconsin 53705, United States; Biophysics Graduate Program, University of Wisconsin-Madison, Madison, Wisconsin 53706, United States; Department of Chemistry, University of Wisconsin-Madison, Madison, Wisconsin 53706, United States; Department of Urology, UT Southwestern Medical Center, Dallas, Texas 75390, United States; Department of Urology, George M. O’Brien Center of Research Excellence, University of Wisconsin-Madison, Madison, Wisconsin 53705, United States; School of Pharmacy, University of Wisconsin-Madison, Madison, Wisconsin 53705, United States; Biophysics Graduate Program and Department of Chemistry, University of Wisconsin-Madison, Madison, Wisconsin 53706, United States; Lachman Institute for Pharmaceutical Development, School of Pharmacy and Wisconsin Center for NanoBioSystems, School of Pharmacy, University of Wisconsin-Madison, Madison, Wisconsin 53705, United States

**Keywords:** benign prostatic hyperplasia, proteomic, glycoproteomic, high throughput

## Abstract

Benign prostatic hyperplasia (BPH) is a disease affecting the majority of aging men; 90% of men develop histological BPH by the time they reach their eighties. BPH can lead to bothersome lower urinary tract symptoms (LUTS), which may reduce quality of life. Many patients fail current treatment options and may progress to surgical intervention. Furthermore, diagnosis is reliant on symptom questionnaires and the cause of LUTS can be difficult to distinguish. Currently, BPH can only be definitively diagnosed through histological analysis of prostate tissue, which is not the standard of care. The resulting lack of clinical tissue samples is a major limitation in investigating disease pathology. Improved understanding of disease development and progression, along with objective biomarkers of disease, is needed for BPH. This investigation uses mass spectrometry (MS)-based proteomics and glycoproteomics to compare healthy prostate tissue with prostate tissue affected by BPH to address this gap in knowledge. By integrating proteomics and glycoproteomics, we identified 206 proteins and 44 glycopeptides that were significantly altered between BPH and control samples. These findings provide deeper insight into disease-associated pathways and may facilitate the identification of clinically relevant targets for further investigation.

## Graphical Abstract

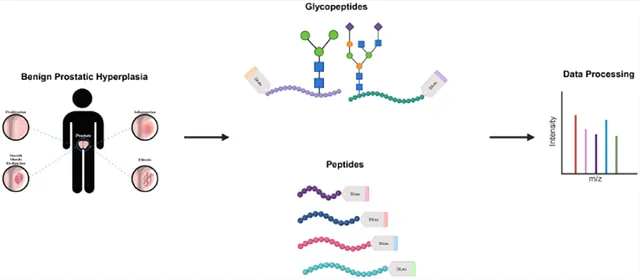

## INTRODUCTION

Benign prostatic hyperplasia (BPH) is a histological condition affecting 90% of men by the time they reach their eighties.^1^ BPH commonly results in lower urinary tract symptoms (LUTS), which may include increased frequency of urination, urgency, nocturia, and incomplete voiding. Men in their 70s experience LUTS twice as much as men in their 50s.^2^ The current gold standard for evaluation of LUTS/BPH is exclusion of other potential underlying causes (e.g., infection, neurologic, etc.) and symptom score questionnaires.^3^ More advanced tools, such as measuring prostate-specific antigen, abdominal imaging, or urodynamic studies, may be used but cannot independently diagnose LUTS/BPH.^3^ LUTS are largely considered to be the relevant clinical manifestation of disease with patient responsiveness to treatment being measured by improvement to symptom indices.^4^ Given that prostate biopsies are not the standard of care, investigation into tissue-specific mechanisms is needed, despite decades of research advancement.

Limited access to clinically relevant prostate specimens is one barrier affecting LUTS/BPH research. Despite being a very common disease in older men, the pathophysiology of LUTS/BPH remains incompletely understood. Although common pathological features, such as epithelial and stromal proliferation, smooth muscle dysfunction, fibrosis, and extracellular matrix (ECM) remodeling, inflammation, and metabolic or mitochondrial dysregulation, have been implicated in disease progression, these processes do not fully explain why many patients fail existing therapies targeting prostate volume (i.e., hyperplasia) or smooth muscle tone.^5–9^ The molecular basis of BPH, particularly at the protein and glycoprotein levels, remains poorly defined.

Protein glycosylation is one of the most common post-translational modifications (PTMs), playing key roles in cell signaling, host–pathogen interaction, protein secretion, protein stability, and the immune response.^10^ Aberrant glycosylation has been implicated in pathological remodeling processes relevant to BPH, including ECM organization and stromal–epithelial signaling, which has been observed in prostate cancer.^11^ Intact glycopeptide analysis provides a powerful means to interrogate site-specific glycosylation and glycan heterogeneity; however, MS-based glycoproteomics faces analytical challenges due to the low abundance and poor ionization efficiency of glycopeptides within complex tissue digests.^12^ Moreover, diagnostic or prognostic biomarkers for BPH remain largely undiscovered.

To overcome these limitations, we employed a highly multiplexed quantitative enrichment workflow integrating global proteomics and N-glycoproteomics in BPH and healthy controls. MS offers a robust platform for studying large-scale glycoprotein regulation across many biological systems.^13–15^ Here, we applied in-house-developed *N,N*-dimethyl leucine (DiLeu) isobaric tags to enable simultaneous, high-throughput quantification of proteomes and hydrophilic interaction liquid chromatography (HILIC) enrichment for glycoproteomes.^16^

Parallel profiling of the proteome and glycoproteome helps distinguish whether changes in glycosylation arise from altered site-specific occupancy or from underlying shifts in protein abundance. To our knowledge, no previous study has jointly characterized the proteomic and N-glycoproteomic landscapes of BPH tissues relative to healthy prostate samples. Our results reveal previously unrecognized alterations in protein expression and N-glycosylation associated with BPH, providing molecular insights that may aid in the development of future diagnostic and therapeutic targets.

## EXPERIMENTAL SECTION

### Chemicals and Materials

Dithiothreitol (DTT) and sequencing grade trypsin were from Promega (Madison, WI). Optima liquid chromatography/mass spectrometry (LC/MS) grade solvents, formic acid (FA), urea, sodium chloride, phosphoric acid, ammonium hydroxide and sodium carbonate were from Fisher Scientific (Pittsburgh, PA). Acetonitrile (ACN), trifluoroacetic acid (TFA), iodoacetamide (IAA), triethylammonium bicarbonate (TEAB), *N,N*-dimethylformamide (DMF), 4-(4,6-dimethoxy-1,3,5-triazin-2-yl)-4-methylmorpholinium tetrafluoroborate (DMTMM) were purchased from Sigma-Aldrich (St Louis, MO). Empty 200 *μ*L TopTips were from Glygen Corp (Columbia, MD). Strong cation exchange (SCX) spin tips were purchased from PolyLC (Columbia, MD). Oasis HLB 1 cc (10 mg) extraction cartridges were purchased from Waters Corporation (Milford, MA). Poly HYDROXYETHYL A bulk material was purchased from PolyLC (Columbia, MD). Protease inhibitor cocktail tablets and phosphatase inhibitor cocktail tablets were from Roche (Mannheim, Germany). All other chemicals and LC-MS grade solvents were purchased from Fisher Scientific (Pittsburgh, PA).

### Specimen Acquisition

Prostate specimens were obtained from organ donors whose families consented at the Southwest Transplant Alliance under IRB-STU112014–033. Organ donors were screened for BPH and excluded from the control cohort if identified. BPH specimens were obtained from patients undergoing simple prostatectomy at UT Southwestern Medical Center. Fresh human tissue samples were dissected into portions for preservation via flash freezing. Flash frozen deidentified samples were provided to the University of Wisconsin–Madison for proteomic analysis. Sample demographics and clinical information are reported in Tables S1 and S2.

### Sample Preparation

Issue samples were homogenized in sodium dodecyl sulfate (SDS) lysis buffer composed of 5% SDS and 50 mM TEAB, with one protease inhibitor tablet and one phosphatase inhibitor tablet added per 10 mL buffer. The lysate was sonicated using a probe sonicator in an ice–water bath at 50% power, with 3-s on/off pulses for two cycles. After sonication, the lysates were centrifuged to keep the supernatant. Proteins were precipitated by adding cold precipitation buffer (ethanol/acetone/acetic acid, 50:49:1, v/v/v) at a ratio of 1:5 (lysate:buffer, v/v), followed by incubation at −20 °C for 18 h. The resulting pellets were obtained by centrifugation, washed twice with cold precipitation buffer, and dissolved in 8 M urea in 50 mM TEAB. Protein concentration was determined using the bicinchoninic acid (BCA) assay. The proteins were reduced with 5 mM DTT at 37 °C for 1 h, followed by alkylation with 15 mM IAA at room temperature in the dark for 30 min. The alkylation was subsequently quenched by the addition of 5 mM DTT for 10 min. The urea concentration was then diluted to 1 M, and proteins were digested with trypsin at a protein-to-enzyme ratio of 100:1 (w/w) at 37 °C for 12 h, followed by a second digestion step at a ratio of 50:1 for 4 h. Subsequently, an additional aliquot of trypsin was added to achieve a final protein-to-enzyme ratio of 50:1, followed by a further 4-h incubation at 37 °C. The digestion was terminated by the addition of TFA to a final concentration of 1%. The samples were stored at −80 °C until future use.

### DiLeu Labeling

The synthesis and labeling of DiLeu tags were performed as previously described.^16–18^ Briefly, DiLeu tags were activated in anhydrous DMF with DMTMM and NMM at 0.6× molar ratios to tags. The mixture was vortexed at room temperature for 1 h, after which the supernatant was added to each peptide sample for labeling. Following an additional 2 h of vortexing at room temperature, the reaction was quenched by adding NH_2_OH to a final concentration of 0.25%. The labeled peptides from all channels were combined, dried under vacuum, and subsequently desalted using SCX spin tips according to the manufacturer’s instructions.

### High pH (HpH) Fractionation

HpH fractionation was performed on a Waters Alliance e2695HPLC using a C18 reversed-phase column (2.1 × 150 mm, 2.5 *μ*m, 100 Å, PolyLC) operating at 0.2 mL/min. Mobile phase A consisted of 10 mM ammonium formate at pH 10 adjusted with ammonium hydroxide and mobile phase B consisted of 90% ACN and 10 mM ammonium formate at pH 10. Peptide separation was achieved using the following gradient: 1% B (0–5 min), 1–40% B (5–50 min), 40–60% B (50–54 min), 60–70% B (54–58 min), and 70–100% B (58–59 min). Fractions were collected every 4 min and nonadjacent fractions were concatenated into six samples before being dried *in vacuo* for LC-MS/MS analysis.

### HILIC Enrichment

Enrichment of DiLeu-labeled glycopeptides was performed using in-house-packed HILIC SPE tips as described previously.^13^ In brief, 3 mg of cotton wool was plugged into an empty TopTip, which was placed on a 2 mL microcentrifuge tube with an adapter unit. After activation in 1% TFA, the material was transferred into the cotton-packed TopTip at a beads-to-peptide ratio of 30:1. The stationary phase was conditioned with 1% TFA and loading buffer (80% ACN/1% TFA) 3 times. The sample was dissolved in loading buffer and loaded onto the HILIC-cotton tip. The tip was centrifuged at 200 g for 2 min, and the flow-through was reloaded to the HILIC-cotton five times to ensure complete retention. The HILIC-cotton tip was washed with loading buffer 6 times. Four fractions were collected: 1) 70% ACN/0.1% FA, 2) 50% ACN/0.1% FA, 3) 25% ACN/0.1% FA, and 4) 0% ACN/0.1% FA. Samples were dried down *in vacuo* before MS analysis.

### NanoLC-MS/MS Analysis

Peptides from each fraction were reconstituted in 0.1% FA and analyzed using a Dionex Ultimate 3000 UPLC coupled to a Thermo Scientific QE-HF mass spectrometer. Peptide separation was achieved on an 18 cm × 75 *μ*m i.d. custom-packed BEH C18 (1.7 *μ*m, 130 Å) capillary column, with a 100 min gradient from 0 to 30% ACN containing 0.1% FA. Full MS scans were acquired over an *m*/*z* range of 300–2000 at a resolution of 60,000, with an automatic gain control (AGC) target of 1E6 and a maximum injection time (IT) of 100 ms. The MS/MS method utilized a top 20 data-dependent acquisition (DDA) mode, with all MS/MS dissociations conducted using a normalized collision energy (NCE) of 30. The MS/MS parameters included a resolution of 60,000, an AGC target of 1E5, and a maximum IT of 200 ms. A dynamic exclusion of 45 s with an isolation window of 1.0 *m*/*z* was applied.

Glycoproteomic analysis was performed using a Fusion Lumos mass spectrometer (Thermo Fisher Scientific, San Jose, CA) coupled with a Dionex Ultimate 3000 UPLC system (Thermo Fisher Scientific, San Jose, CA). Samples were reconstituted in 0.1% FA and separated on the same column as above using a gradient from 3% to 40% ACN in 0.1% FA over 100 min, at a flow rate of 0.3 *μ*L/min. Data acquisition was carried out in top-speed mode with a 3-s cycle time. Full MS scans were collected over an *m*/*z* range of 400–2000 at a resolution of 60,000. Precursor ions underwent higher-energy C-trap dissociation (HCD) with an NCE of 30, incorporating a ±3% stepped HCD collision energy. Tandem MS spectra were acquired at a resolution of 60,000 with a lower mass limit of *m*/*z* 110. Each sample was acquired in technical replicates.

### Data Analysis

Intact N-glycopeptide data were processed using Byonic (version 2.9.38, Protein Metrics Inc., San Carlos, CA) integrated within Proteome Discoverer 2.5 (Thermo Fisher Scientific). Raw files were searched against UniProt *Homo sapiens* reviewed database (December 2023). Trypsin was selected as the enzyme and two maximum missed cleavages were allowed. Searches were performed with a precursor mass tolerance of 10 ppm and a fragment mass tolerance of 0.02 Da. Fixed modifications were specified as carbamidomethylation (+57.02146 Da) on cysteine residues and DiLeu (+145.12801 Da) on peptide N-terminus and lysine residues. Dynamic modifications included oxidation of methionine (+15.99492 Da), deamidation on asparagine and glutamine (+0.984016 Da), and N-glycosylation. Glycan modifications were searched against a human glycan database expanded from Byonic embedded N-glycan database. Peptide identification results were filtered at Byonic score > 150, PEP 2D < 0.05, |Log Prob| > 1. The total proteome data was used to normalize the enriched glycopeptides. For protein-level normalization of glycopeptide abundance, normalized log_2_ glycopeptide intensities were matched to their corresponding protein groups. The log_2_ protein abundance was then subtracted from the log_2_ glycopeptide abundance for each sample, generating a protein-normalized glycoform abundance value that reflects relative glycosylation independent of changes in total protein expression. Proteomic data were analyzed using the same parameters, except that searches were conducted with the Sequest HT search engine and without dynamic N-glycosylation modifications. All other parameters were kept at their default settings. Quantitative data were processed in Perseus (version 2.1.3.0).^19^ Missing values were handled using imputation based on the Gaussian distribution. Differential abundance was assessed using two-sided Student’s *t* test, and permutation-based false discovery rates (FDR) 5% was applied. Bioinformatics analysis, including hierarchical clustering and volcano plots, was conducted using in-house Python, R scripts, and an online platform https://www.bioinformatics.com.cn.^20^ The MS data have been deposited to the ProteomeXchange Consortium via the MassIVE partner repository with the accession number MSV000100344.

## RESULTS AND DISCUSSION

### Experimental Design: Highly Multiplexed Quantification of Protein Expression and PTMs Was Performed across Large Data Sets

**Figure 1.**
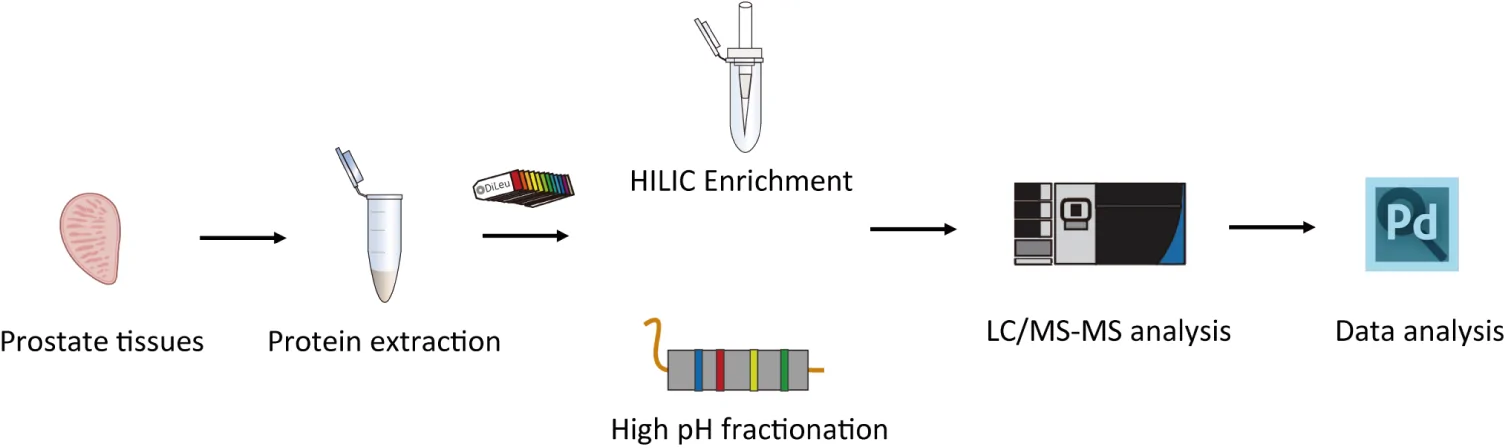
Experimental workflow of quantitative analysis using DiLeu isobaric labeling strategy. Proteins from prostate tissues were extracted using sonication (n = 7, each) and digested into peptides. DiLeu isobaric labeling was used before HILIC enrichment and HpH fractionation to achieve quantitative glycoproteomics and proteomics analysis. The samples were analyzed using LC-MS/MS, and data was processed with Proteome Discoverer and Byonic.

As shown in Figure 1, isobaric labeling of proteins from prostate tissues with 8-plex DiLeu tags was employed for quantitative (glyco)proteomic analysis. BPH tissues were analyzed as cases, while healthy prostate tissues from organ donors served as controls. Each experimental group (control and BPH) included seven biological replicates (n = 7). Samples were processed and analyzed using the same workflow. As shown in Supplemental Table 1, donor controls were significantly younger (mean age 33.1) than BPH patients (mean age 69.4). Given BPH is an age-associated disease, this is an expected limitation of the analysis. Furthermore, other demographic variables, such as self-reported ethnicity and body mass index (BMI) were well matched. While BPH is a heterogeneous disease, BPH samples in this study came from men with large prostates and severe LUTS. The 8-plex DiLeu tags were strategically distributed across two labeling sets to avoid quantification bias caused by deuterium-induced retention time shifts during LC separation. After labeling, the DiLeu-tagged tryptic digest from each set was split into two parallel experiments targeting the proteome and glycoproteome, respectively. For glycoproteomic analysis, one portion was subjected to HILIC enrichment to selectively capture glycopeptides, which were then analyzed by LC–MS/MS for identification and relative quantification. The remaining nonenriched portion was processed through SCX cleanup to remove residual labeling reagents, followed by high-pH fractionation to reduce sample complexity prior to LC–MS/MS analysis for global proteome profiling. This experimental design enables highly multiplexed and reproducible quantification of both protein expression and PTMs across large biological data sets. The use of DiLeu isobaric tags offers high labeling efficiency and minimizes interbatch variability, facilitating accurate comparison between disease and control groups. Parallel analysis of the proteome and glycoproteome allows simultaneous assessment of protein abundance and glycosylation dynamics within the same biological system, providing complementary insights into molecular alterations associated with BPH pathogenesis.

### Quantitative Analysis of Significantly Altered Proteomic and Glycoproteomic Features in BPH

**Figure 2.**
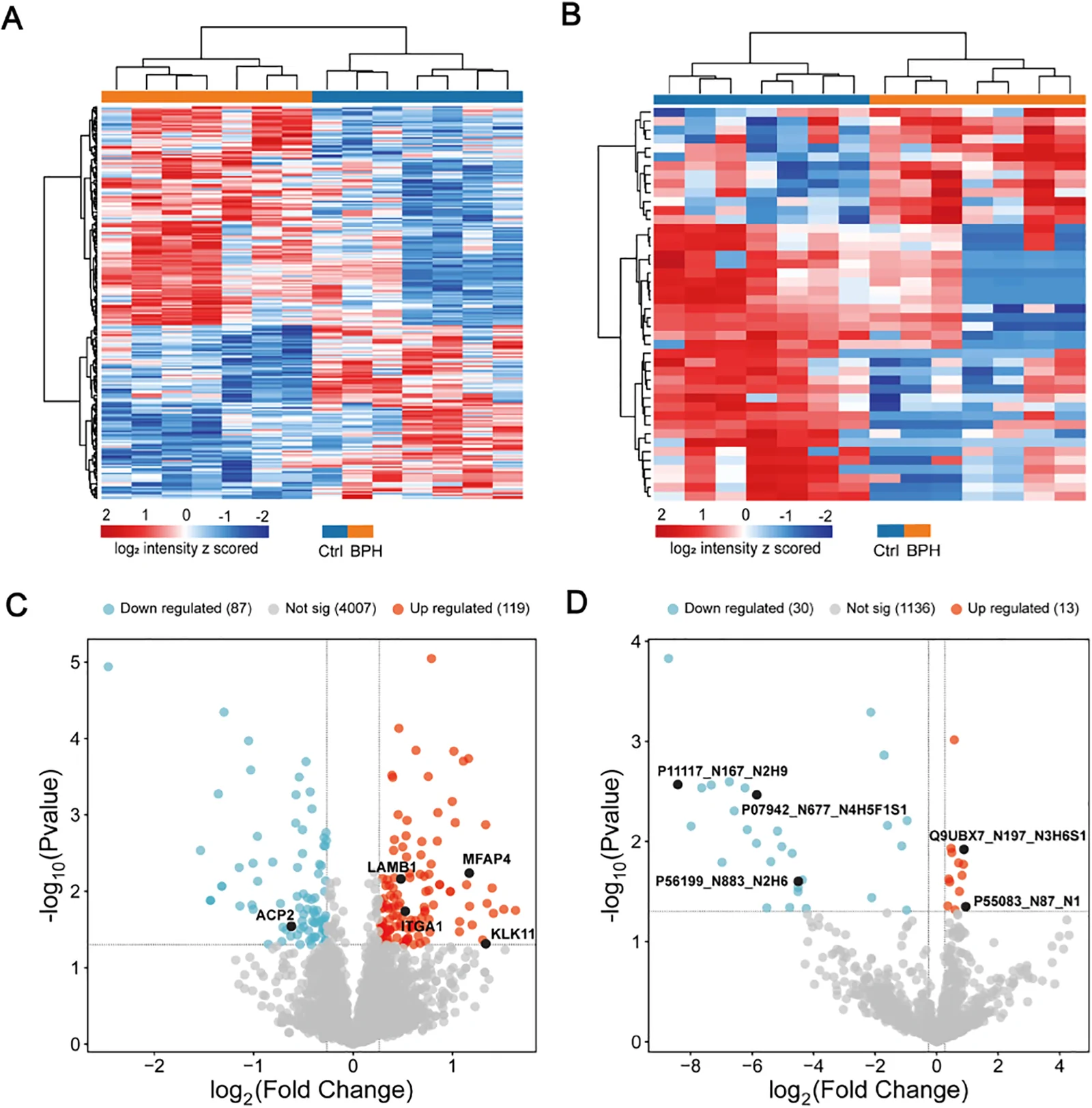
Quantitative analysis of proteomic and glycoproteomic alterations in prostate tissues from BPH and donor controls. Hierarchical clustering analysis of DiLeu reporter ion intensities of all quantified proteins (A) and glycopeptides (B) in BPH and control groups. Volcano plots show pairwise comparisons of peptide (C) and glycopeptide (D) expression levels between BPH and control groups. Points above the dashed lines represent significantly altered peptides or glycopeptides (two-sided *t* test, *p*-value < 0.05, fold change threshold is 1.2).

Comprehensive proteomic and glycoproteomic profiling was conducted to investigate molecular alterations associated with BPH. We performed hierarchical clustering analysis of significantly altered peptides and glycopeptides to explore their expression patterns across experimental groups (Figure 2A,B). The heatmaps show column-wise clustering of biological replicates in control and BPH groups, revealing greater intergroup differences than intragroup variations and underscoring reproducible proteomic and glycoproteomic remodeling in BPH tissues.

**Figure 3.**
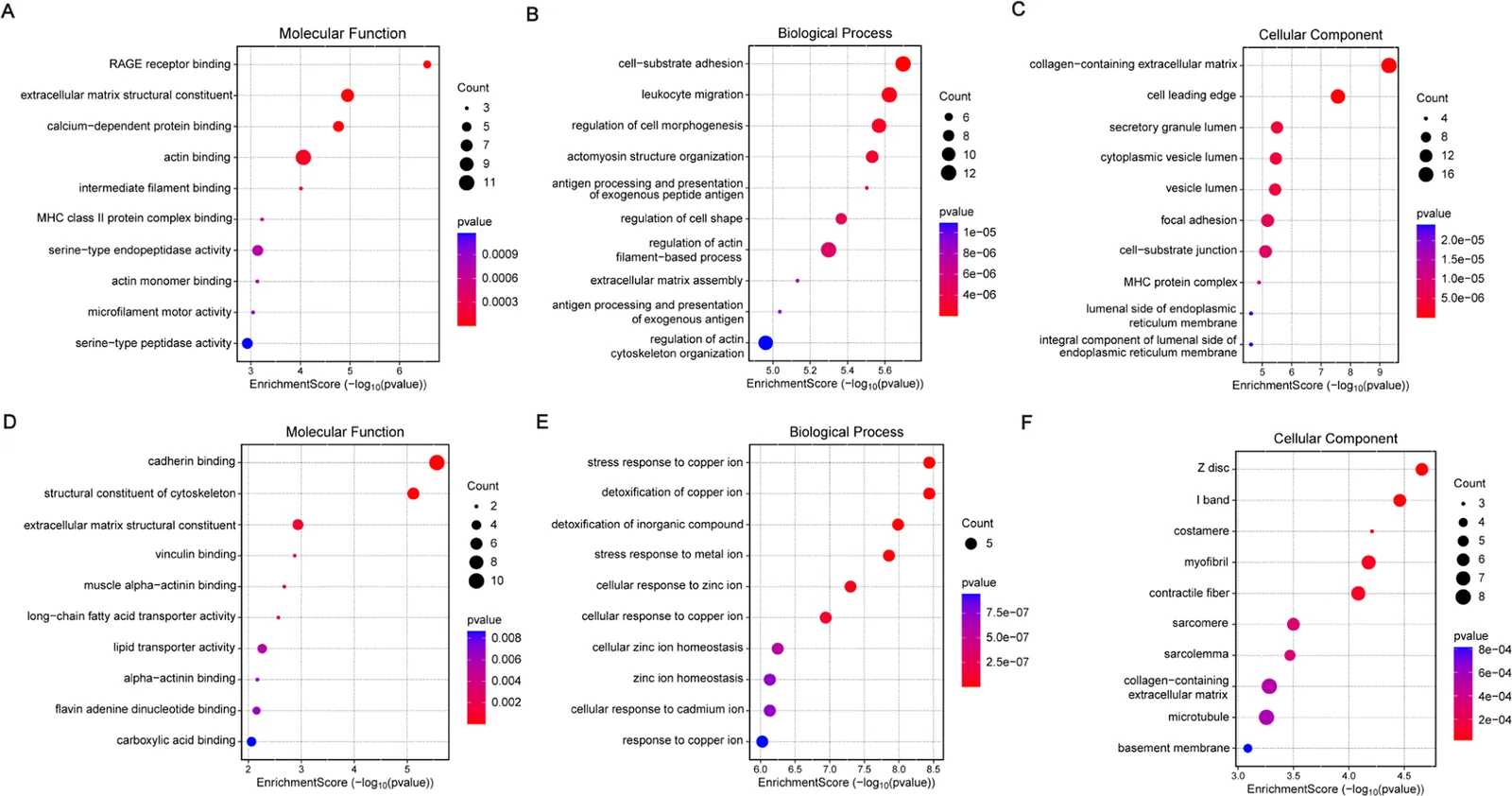
GO analysis of significantly upregulated proteins based on (A) biological process, (B) cellular component, and (C) molecular function, and significantly downregulated proteins based on (D) biological process, (E) cellular component, and (F) molecular function. The top 10 most significant categories are shown.

To enable robust pairwise comparisons between groups, we performed Student’s *t* tests and further filtered the data using a fold change greater than 1.2 and a p-value smaller than 0.05. A total of 206 proteins were identified as significantly altered (Figure 2C, Table S3). Notably, COL28A1 was significantly decreased in BPH tissues, suggesting selective remodeling of the extracellular matrix (ECM). As a nonfibrillar collagen associated with basement membrane structure and cell–matrix adhesion, reduced COL28A1 expression may reflect disruption of epithelial–stromal architecture or a shift toward a fibrotic remodeling.^21^ This loss may contribute to altered mechanobiology and stromal signaling characteristic of BPH progression. Gene Ontology (GO) analysis revealed differentially enriched pathways in BPH (Figure 3A–F). Among significantly upregulated proteins, ECM components, particularly collagens and cytoskeletal elements, were prominently enriched (Figure 3A,B). Cellular component analysis similarly highlighted enrichment of the ECM and focal adhesions, both of which have been implicated in BPH pathology (Figure 3C). These findings are consistent with the established roles of fibrosis in BPH progression^7^ and emerging evidence linking cytoskeletal remodeling to prostatic hyperplasia.^22^ Immune-related pathways were also upregulated, consistent with the known contribution of inflammation to BPH development.^23^ Distinct enrichment patterns were also observed among downregulated proteins. Several pathways associated with reduced protein abundance mapped to the ECM (Figure 3D–F), consistent with altered epithelial–stromal junctions and ECM remodeling in BPH.^24^ Interestingly, the majority of biological processes downregulated in BPH were related to heavy metal metabolism (Figure 3E). Closer examination revealed that many of these proteins are involved in antioxidant responses, aligning with previous reports of oxidative stress observed in BPH.^25,26^ Additionally, lipid and long-chain fatty acid transport pathways were significantly downregulated (Figure 3D). While the role of fatty acid metabolism in BPH remains poorly understood, oleic acid has been shown to improve urinary voiding function in mouse models of dysfunction,^9^ suggesting a potential avenue for future therapeutic investigation.

In parallel, a total of 44 glycopeptides were found to be significantly altered, with 13 upregulated and 31 downregulated in BPH (Figure 2D, Table S4). The majority of dysregulated glycoproteins were localized to the extracellular region (Figure 5C), in accordance with the fact that most N-glycoproteins are secreted or membrane-bound and play key role in extracellular matrix remodeling and cell–cell signaling.^27,28^ Collectively, the proteomic and glycoproteomic data sets reveal coordinated yet distinct regulation of protein abundance and glycosylation in BPH. This integrative strategy highlights the analytical advantage of combining global and site-specific quantification to uncover functionally relevant molecular alterations in the prostate.

### Differential Expression of Proteins and Glycoproteins and Their Relevance to BPH

**Figure 4.**
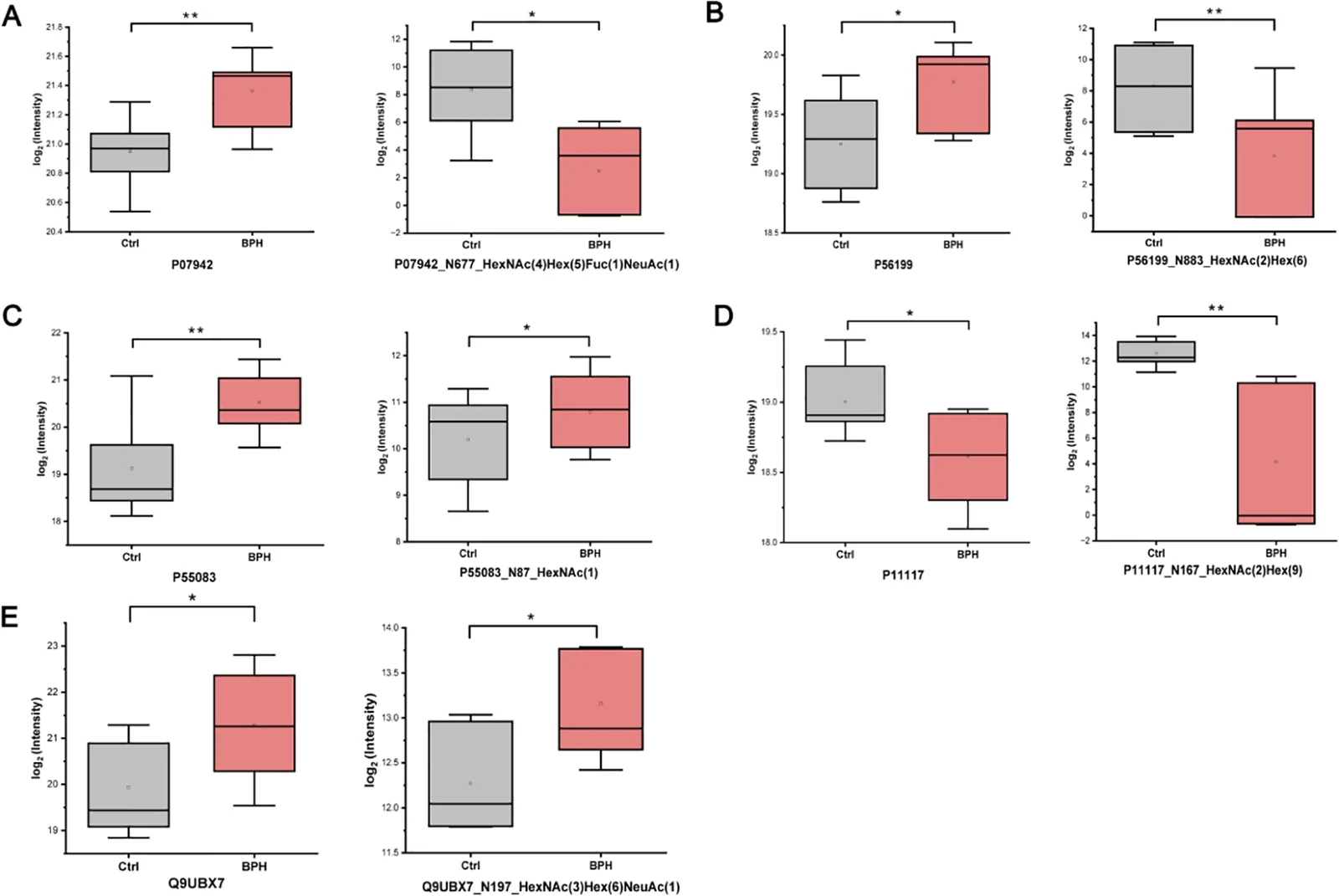
Differential expression of proteins and corresponding glycoforms in control and BPH tissues. Box plots show log2 intensity values for proteins (left) and matched glycoforms (right) in Ctrl and BPH samples. (A) P07942 increased, while its glycoform decreased in BPH. (B) P56199 increased, while its glycoform decreased. (C) P55083 and its glycoform both increased. (D) P11117 and its glycoform both decreased. (E) Q9UBX7 and its glycoform both increased. Boxes indicate interquartile range, center lines indicate median, and whiskers indicate range. *p*-value < 0.05 (*), *p*-value < 0.01 (**).

Differential expression of specific proteins in the global proteome and glycoproteome can provide insight into broader disease processes. Here, we highlight five proteins that were significantly different between BPH and control tissues in untargeted proteomics analysis and that also exhibited at least one significantly altered glycoform. Figure 4A shows that laminin subunit beta-1 (LAMB1, P07942) is significantly increased in BPH tissue, whereas the glycoform with a modification at residue N677 is decreased in BPH. LAMB1 plays a key role in ECM organization and epithelial cell proliferation. Previous studies have identified LAMB1 as a marker of BPH; for example, analysis using patient-derived xenografts showed a progressive increase in LAMB1 abundance in BPH grafts over time.^29^ Other proteomic investigations have also highlighted the potential of LAMB1 as a serum biomarker for distinguishing prostate cancer from benign prostate disease.^30^

Figure 4B highlights another ECM-associated protein altered in BPH, integrin alpha-1 (ITGA1, P56199). ITGA1 functions as a receptor for collagens, laminins, and various cytokines. While overall ITGA1 protein expression is increased in BPH, glycosylation at the N883 site is reduced in BPH. Elevated ITGA1 expression has been linked to cancer, type 2 diabetes, and cardiovascular disease. Although glycosylation is known to influence ITGA1 function, relatively little is understood about the specific role of the N883 glycosylation site.^31–33^

Microfibril-associated glycoprotein 4 (MFAP4, P55083) is another ECM protein that is upregulated in BPH, along with its N87 glycoform (Figure 4C). Previous studies have identified MFAP4 gene expression in both mouse and human prostate tissue and suggest that it may serve as a marker of interstitial fibroblasts.^34^ In murine models of BPH, this fibroblast population has been proposed to primarily contribute to matrix adhesion.^35^ Beyond its identification as a cell-type marker, however, little is known about the functional role of MFAP4 in BPH disease progression.

Lysosomal acid phosphatase (ACP2, P11117) is a metabolic enzyme that regulates intracellular phosphate availability, thereby influencing ATP metabolism. Both ACP2 protein abundance and its N167 glycoform are decreased in BPH compared to donor controls (Figure 4D). In a previous proteomic study, ACP2 levels were also reported to be reduced in the urine of BPH patients relative to that of prostate cancer patients.^36^ Although limited information is available regarding the role of ACP2 specifically in the prostate, dysregulation of ACP2 has been associated with various malignancies, Parkinson’s disease, and infectious conditions. Given the reported importance of mitochondrial function in BPH,^9,37^ alterations in ACP2 may indirectly affect cellular energy homeostasis and contribute to disease progression.

Finally, Kallikrein-11 (KLK11, Q9UBX7) and its N197 glycoform are both significantly increased in BPH tissue (Figure 4E). Kallikrein proteins are a family of serine proteases with diverse biological functions and have been widely investigated as disease biomarkers, particularly in cancer. One study of prostate cancer samples reported reduced KLK11 levels in more aggressive tumors, suggesting that KLK11 expression may help distinguish nonmalignant prostatic hyperplasia from malignant prostate cancer proliferation.^38^ The functional significance of the N197 glycoform; however, remains poorly understood.

### Glycosylation Heterogeneity in BPH

**Figure 5.**
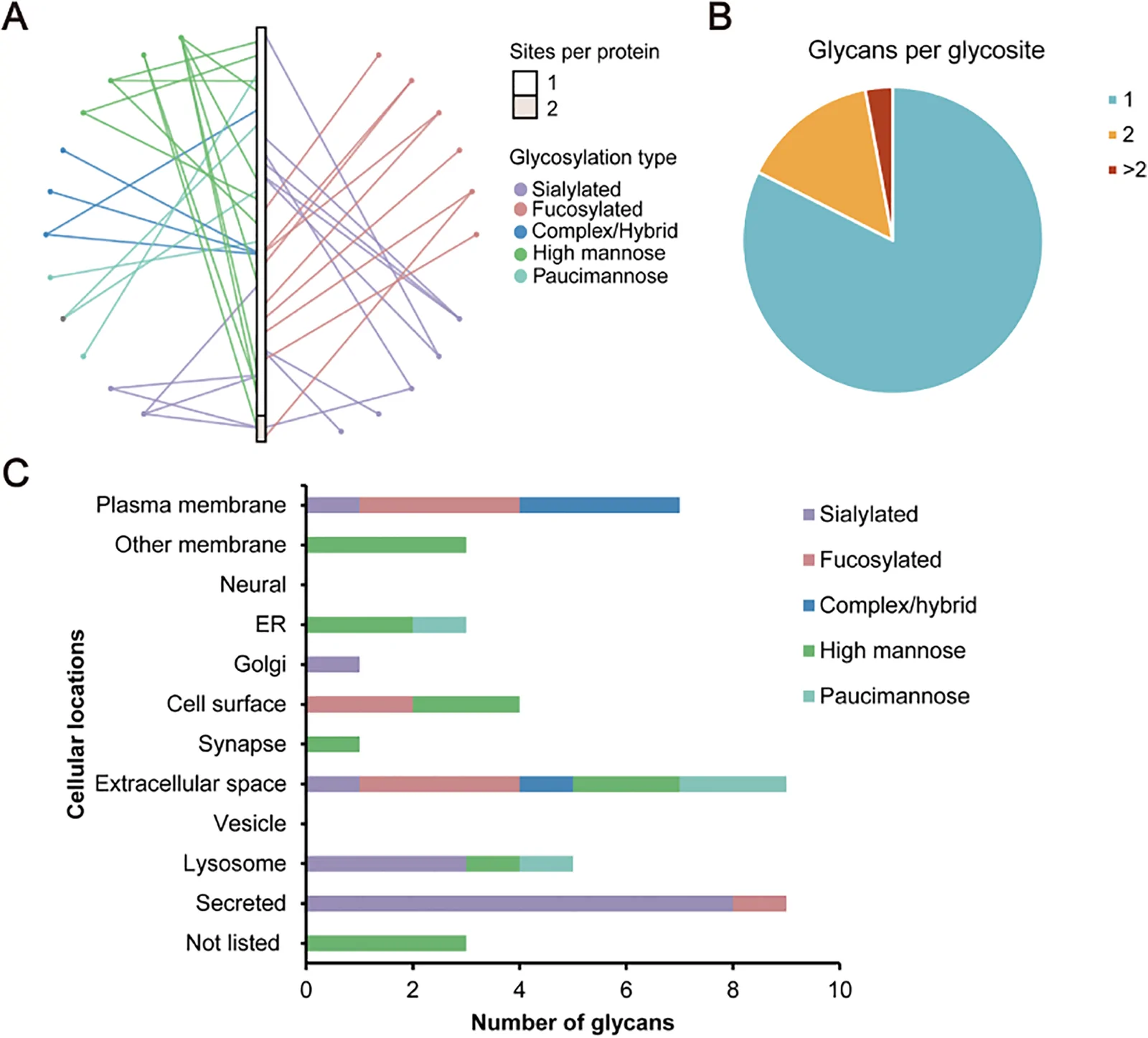
Glycosylation heterogeneity in BPH. (A) Glycoprotein–glycan network diagram mapping which glycans (outer nodes) modify which proteins (inner bars). Glycoproteins are sorted by the number of glycosylation sites. Glycan nodes and their links to proteins are colored according to glycosylation type. (B) Significantly altered glycans per glycosylation site identified in prostate tissues. (C) Distribution of glycosylation according to subcellular localization, as defined by GO cellular component terms.

To delineate N-glycosylation heterogeneity in BPH, we profiled significantly altered site-specific glycoforms and glycosylation site patterns in human prostatic tissues. As shown in Figure 5, glycoproteins exhibited substantial heterogeneity in both glycosylation site occupancy and glycan composition. Figure 5A depicts a glycoprotein-glycan network of significantly altered glycopeptides. Nearly 88% of glycoproteins were observed to contain a single glycosylation site, whereas 12% of glycosites were modified by more than one glycan. Furthermore, 3% of glycosites carried more than two distinct glycans, indicating a high degree of glycosylation heterogeneity in prostate tissues (Figure 5B). This structural diversity reflects dynamic regulation of glycan processing in the Golgi apparatus and endoplasmic reticulum (ER), where branching, elongation, and terminal modifications are finely tuned by glycosyltransferases and glycosidases.^39^ Among all significantly altered glycopeptides, 30% and 21% were sialylated and fucosylated, respectively (Figure 5A). Two upregulated sialylated glycopeptides were observed at a specific site of lysosome-associated membrane glycoprotein 1, a protein critical for vesicular trafficking and lysosome function. Increased terminal sialylation can modulate receptor signaling and promote immune evasion by masking galectin-binding sites,^40^ whereas core fucosylation is known to enhance growth factor receptor activation and stromal proliferation.^10,41^ Both glycosylation features are relevant to disease progression in BPH. In addition, detection of high-mannose and paucimannose glycans on ER- and vesicle-localized proteins may suggest altered protein quality control or stress-associated accumulation of immature glycoproteins–mechanisms that have not been extensively investigated in BPH.^42^

Subcellular localization analysis revealed that heterogeneous glycosylation was predominantly associated with plasma membrane, secreted, and extracellular proteins (Figure 5C). Several ECM-associated glycoproteins, including laminins, collagens, and integrins, exhibited multiple glycosylation sites decorated with distinct glycan types, suggesting that local microheterogeneity may influence protein folding, secretion, and intermolecular interactions.^43,44^ While the mechanistic contribution of aberrant glycosylation is outside the scope of this investigation, it does further support existing findings demonstrating the clinical relevance of the glycoproteome in BPH. For example, previous investigations demonstrated that protein glycoforms in the serum were useful in distinguishing between BPH and prostate cancer, where prostate specific antigen, the current clinical gold standard, was insufficient.^45,46^ Given there remains no noninvasive test for diagnosing BPH, further studies should build on existing work to leverage changes to glycoforms between healthy prostate, BPH, and prostate cancer for effective diagnostic biomarkers. Existing publications have primarily focused on comparing the glycoproteome of BPH and prostate cancer, leaving a gap in our understanding of the glycoproteome of the healthy prostate, which is included in this study. Thus, we observed substantial glycosylation heterogeneity within the BPH proteome, characterized by pronounced site-specific and subcellular variation that likely arises from dysregulated glycan processing and secretory pathway function. Such alterations in glycoprotein structure and spatial distribution may reshape the extracellular microenvironment, modulate epithelial–stromal communication, and ultimately contribute to tissue enlargement and pathological remodeling in BPH.

## CONCLUSIONS

In this study, we applied a highly multiplexed DiLeu-based quantitative workflow to simultaneously profile the proteome and N-glycoproteome of human BPH and healthy prostate tissues. The strengths of this investigation include parallel quantification of protein abundance and site-specific glycosylation and the use of clinically relevant tissues for both healthy controls and BPH specimens. To date, the majority of investigations for both proteomics and glycoproteomics in BPH have been compared to prostate cancer specimens, creating a gap in our understanding of the healthy prostate we aimed to address. We also acknowledge several limitations of the study, including a relatively small sample size, a significant age difference between controls and BPH patients, and a focus on large prostates, which is not fully representative of BPH. Overall, these findings revealed coordinated yet distinct alterations in ECM organization, cytoskeletal remodeling, immune activity, and metabolic pathways. Integration of quantitative proteomics and glycoproteomics enabled discrimination between changes driven by differential protein expression and those arising from altered glycan site occupancy or composition. Proteins such as LAMB1, ITGA1 and KLK11 exhibited discordant regulation between global expression and individual glycoforms, underscoring the functional importance of glycosylation microheterogeneity in BPH. Downregulation of antioxidant-related pathways and perturbations in lipid transport further suggest disease-relevant metabolic axes not captured by transcript-level analysis alone. Collectively, this work provides the first integrated proteomic and N-glycoproteomic atlas of BPH tissues relative to healthy controls, enabled by high-throughput isobaric labeling and comprehensive LC–MS/MS analysis. These findings highlight the value of combining multiplexed quantitative proteomics with site-specific glycoproteomic profiling to elucidate complex molecular processes underlying benign urologic disease.

## Supplementary Material

Supplemental Information 1 Supplemental experimental section; Table S1: sample demographics for organ donor controls and BPH cases; Table S2: clinical characteristics of BPH cases (PDF)

Supplemental Information 2 Table S3: significantly changed proteins identified from donor controls and BPH samples (separate Excel spreadsheet) (XLSX)

Supplemental Information 3 Table S4: significantly changed glycopeptides identified from donor controls and BPH samples (XLSX)

The Supporting Information is available free of charge at https://pubs.acs.org/doi/10.1021/jasms.6c00143.
