# Supplementary material for: High-Throughput Proteomic and Glycoproteomic Analyses in Benign Prostatic Hyperplasia
Source: J Am Soc Mass Spectrom. Author manuscript; Available in PMC 2026 Sep 13. (PMC13570878; doi:10.1021/jasms.6c00143)
Supplement: Supplementary file 1 [file NIHMS2208743-supplement-Supplemental_Information_1.pdf]

Supplemental Information

High-Throughput Proteomic and Glycoproteomic Analyses in Benign Prostatic Hyperplasia

Feixuan Wu[1#], Alexis E. Adrian[2#], Allen Zhao[3], Han Zhang[1], Peng-Kai Liu[4], Yajing Lu[5], Douglas W. Strand[6], William A. Ricke[2], Lingjun Li[1,4,5,7,8*]*

[1]School of Pharmacy, University of Wisconsin-Madison, WI 53705, USA. [2]Department of Urology, George M. O'Brien Center of Research Excellence, University of Wisconsin-Madison, WI 53705, USA. [3]Department of Biochemistry, University of Wisconsin-Madison; [4]Biophysics Graduate Program, University of Wisconsin-Madison, WI 53706, USA. [5]Department of Chemistry, University of Wisconsin-Madison, WI 53706, USA. [6]Department of Urology, UT Southwestern Medical Center, TX 75390, USA. [7]Lachman Institute for Pharmaceutical Development, School of Pharmacy, University of Wisconsin-Madison, Madison, WI 53705, USA. [8]Wisconsin Center for NanoBioSystems, School of Pharmacy, University of Wisconsin-Madison, Madison, WI 53705, USA.

Downloaded from pubs.acs.org/jamsef/article-supplement/5243703/pdf/js6c00143_si_001 by guest on 03 September 2026

Table of Contents

Table S1. Sample demographics for organ donor controls and BPH cases.

Table S2. Clinical characteristics of BPH cases.

Table S3. Significantly changed proteins identified from donor controls and BPH samples (separate excel spreadsheet).

Table S4. Significantly changed glycopeptides identified from donor controls and BPH samples (separate excel spreadsheet).

Downloaded from pubs.acs.org/jamsef/article-supplement/5243703/pdf/js6c00143_si_001/ by guest on 03 September 2026

	Donor (n=7)	BPH (n=7)
Age	33.1 (5.1)	69.4 (4.4)
Body Mass Index (BMI)	28.4 (2.6)	28.3 (4.1)
**Ethnicity**		
White	3	6
Black	2	0
Hispanic/Latino	2	1

Table S1: Sample demographics for organ donor controls and BPH cases. Age and Body Mass Index are reported as mean (standard deviation). Ethnicity is reported as number of individuals who self-identified as such.

Metric (BPH Only)	Mean (SD)	Sample Size
Flow Rate	8.1 (5.9)	4
Post Void Residual	116.4 (203.5)	5
Prostate Volume	108.9 (30.5)	7
PSA	7.4 (4.4)	5
AUASS	23.4 (6.7)	5
	**N (%)**	
History of Urinary Retention?	4 (57.1%)	7
History of 5ARI use?	3 (42.9%)	7
History of α-blocker use?	3 (42.9%)	7

Table S2: Clinical characteristics of BPH cases.

Downloaded from pubs.acs.org/jamsef/article-supplement/5243703/pdf/js6c00143_si_001 by guest on 03 September 2026
